# Inguinal Hernia Waiting Lists; Medical and Financial Implications

**Published:** 1989-11

**Authors:** A. H. Davies, J. Mountfield, C. P. Armstrong

**Affiliations:** Surgical Senior House Officer; Surgical House Officer; Surgical Senior Registrar, Department of Surgery, Bristol Royal Infirmary, Bristol. BS2 8HW

**Keywords:** Waiting list, herniorraphy


Bristol Medico-Chirurgical Journal Volume 104 (iv) November 1989

### Inguinal Hernia Waiting Lists: Medical and Financial Implications

**A. H. Davies, M.A., F.R.C.S.,***
Surgical Senior House Officer

**J. Mountfield, M.B., B.Ch.,**
Surgical House Officer

**C. P. Armstrong, M.D. F.R.C.S.,**
Surgical Senior Registrar, Department of Surgery, Bristol Royal Infirmary, Bristol. BS2 8HW

* Correspondence to: Mr. A. H. Davies, Department of Urology, Churchill Hospital, Headington, Oxford. OX3 7LF

**Key words:** Waiting list, herniorraphy.

In two years, 62 patients presented acutely with symptoms secondary to an inguinal hernia. Nine (15%) were already on the waiting list for herniorraphy. On projected figures of bed occupancy alone these patients cost the N.H.S. at least £750 more per patient than those admitted for an elective repair. The medical implications were a minium of a 12 fold increase in postoperative complications in the emergency cases. The above emphasizes the need to keep the waiting list for herniorraphy down to a minimum.

### INTRODUCTION

Over a thousand people die per annum in England and Wales as a result of an inguinal hernia. The operation of elective inguinal herniorraphy has minimal mortality and morbidity whether performed as a day case or as a formal inpatient procedure. Inguinal herniorraphy is often regarded as a minor procedure and consequently is given little priority on general surgical waiting lists. Results show that there is increased morbidity and mortality from the emergency treatment of inguinal hernias 2, 3. The question therefore arises as to the medical and financial implications of long waiting lists for inguinal herniorraphy.

### PATIENTS, METHODS AND RESULTS

Over a two year period 62 patients (55 men and 7 women) with a median age of 66 years (20–94) were admitted acutely with an inguinal hernia. These were compared to age and sex matched patients undergoing elective surgery in the same period on two general surgical firms "A" and "B" in the same hospital. Table 1 illustrates the comparison between the acute admissions already on a surgical waiting list and the elective practice on firms A and B with regard to length of admission, complication rate and length of time on waiting list. Table 2 shows the complications found in all the elective patients compared to all the emergency cases. Nine patients (15%) from the acute admission group were on a waiting list, their median time of hospital stay was 16 days (5–90), one of these patients died postoperatively after a myocardial infarct. Whereas the median time of hospital stay for the remaining acute admissions was 7 days (2–26). In comparison to the median time of stay for routine surgery on average an extra 5 days were required for each acute admission and in the case of those patients already on a waiting list an extra 12 days were required. One patient in the latter group was in hospital for 90 days, if he is excluded the median time for the rest of the group was 7 days, which is the same value as for the acute admissions not on a waiting list. The cost of bed occupancy for a 24 hour period in the N.H.S. is approximately £150 (dependent on hospital). Therefore the cost per patient on a waiting list needing acute intervention was an extra £1800, if one excludes our patient admitted for 90 days, a corrected figure would be an extra £750. On top of this figure is the expenditure involved in treating the postoperative complications which in this study included two patients who required dialysis for renal failure.

**Table I**

**Illustrates the time spent on the waiting list and the complication rate for the routine admissions on two seperate general surgical firms compared with those emergency cases already on a waiting list**

| | *Emergency Admission on waiting list* | *Emergency Admission not on waiting list* | *Firm A Admission* | *Firm B Admission* |
|---|---|---|---|---|
| n | 9 | 53 | 62 | 62 |
| Median time on waiting list (range) months | 9 (1–30) | / | 6 (2–14) | 7 (1–26) |
| Median duration of hospital stay (range) days | 16 (5–90) | 7 (2–26) | 4 (1–13) | 4 (2–7) |
| Complication rate | 55.6% | 35.8% | 3.2% | 1.6% |

**Table II**

**Complications of inguinal herniorraphy**

| *Emergency* | | *Firm A* | | *Firm B* | |
|---|---|---|---|---|---|
| Urinary retention | 4 | Urinary retention | 1 | Urinary retention | 1 |
| (T.U.R.P.) | 2 | | | | |
| Scrotal haematoma | 5 | Scrotal haematoma | 1 | | |
| Renal Failure | 2 | | | | |
| Orchiectomy | 3 | | | | |
| Pneumonia | 4 | | | | |
| Cardiac | 4 | | | | |
| (Death | 1 | | | | |
| Urinary tract infection | 1 | | | | |
| Small bowel infarct post surgery | 1 | | | | |

continued on p 106

### REFERENCES

INGUINAL HERNIA WAITING LISTS
continued from p 104

### DISCUSSION

These results confirm the increased morbidity and mortality of surgery and the greater financial cost to the N.H.S. for the emergency treatment of inguinal hernias. The recent C.E.P.O.D 3 report showed that 59.2% of deaths due to inguinal hernias were in patients presenting as an acute emergency and in that study half the deaths were potentially preventable.

In this country 17.3% of inguinal hernias present as an emergency 4. By extrapolating from our figures above this means that nearly 1 in 5 inguinal henia repairs costs at least £750 more than if admitted electively. Furthermore the cost of treating the resultant complications adds even more expense.

This study shows that surgically treating patients with inguinal hernias as rapidly as possible is desirable from both a medical and financial viewpoint. The long waiting lists for inguinal herniorraphy in this country need to be urgently reduced, this could be achieved by increasing the facilities for day case surgery 5.

### REFERENCES

1. Mortality statistics: cause 1985. O.P.C.S. Series D.H.2 No. 12 H.M.S.O. 1987 34–108.
2. O'HIGGINS, N. (1984) Hernia. In Surgical Management (Ed Taylor S., Chisholm G. D., O'Higgins N., Shields R.) London: William Hieneman Medical Books 812–28.
3. BUCK, N., DEVLIN, H.B., LUNN, J.N. (1988) Confidential Enquiry into Perioperative Deaths. 51–52.
4. Hospital in patient enquiry 1985. (1987) O.P.C.S. D.H.S.S. H.M.S.O. 63.
5. DAVIES, A. H., HORROCKS, M. Patient evaluation and complications of inguinal herniophy under local anaesthetic. *J. R. Coll Surg Edin* **34**, 1989 137–9.

